# False-Belief Understanding and Language Ability Mediate the Relationship between Emotion Comprehension and Prosocial Orientation in Preschoolers

**DOI:** 10.3389/fpsyg.2016.01534

**Published:** 2016-10-07

**Authors:** Veronica Ornaghi, Alessandro Pepe, Ilaria Grazzani

**Affiliations:** ^1^Department of Human Sciences and Education, University of Milano-BicoccaMilan, Italy; ^2^Department of Psychology, University of Milano-BicoccaMilan, Italy

**Keywords:** emotion comprehension, prosocial orientation, theory of mind, language, false-belief understanding

## Abstract

Emotion comprehension (EC) is known to be a key correlate and predictor of prosociality from early childhood. In the present study, we examined this relationship within the broad theoretical construct of social understanding which includes a number of socio-emotional skills, as well as cognitive and linguistic abilities. Theory of mind, especially false-belief understanding, has been found to be positively correlated with both EC and prosocial orientation. Similarly, language ability is known to play a key role in children’s socio-emotional development. The combined contribution of false-belief understanding and language to explaining the relationship between EC and prosociality has yet to be investigated. Thus, in the current study, we conducted an in-depth exploration of how preschoolers’ false-belief understanding and language ability each contribute to modeling the relationship between children’s comprehension of emotion and their disposition to act prosocially toward others, after controlling for age and gender. Participants were 101 4- to 6-year-old children (54% boys), who were administered measures of language ability, false-belief understanding, EC and prosocial orientation. Multiple mediation analysis of the data suggested that false-belief understanding and language ability jointly and fully mediated the effect of preschoolers’ EC on their prosocial orientation. Analysis of covariates revealed that gender exerted no statistically significant effect, while age had a trivial positive effect. Theoretical and practical implications of the findings are discussed.

## Introduction

In the research domain of children’s socio-emotional competence, a body of empirical evidence suggests that children’s understanding of their own and others’ emotions predicts their level of prosocial behavior toward peers and adults (Cassidy et al., 2003; Denham et al., 2003; Ensor and Hughes, 2005; Garner et al., 2008). In the present work, we set out to advance our knowledge of this relationship with respect to the broader theoretical construct of social understanding, which encompasses a wide range of abilities helping children to be aware of their own and others’ inner states, from understanding of emotions and feelings (emotion comprehension, EC) to appreciating intentions, desires and beliefs (theory of mind, ToM). Social understanding also includes the ability to understand the responses and actions of others, and to be effective in one’s social and communicative interaction (Carpendale and Lewis, 2006; Hughes, 2011).

While the existing literature includes studies on the independent contributions of EC and ToM to explaining the development of prosociality (e.g., Walker, 2005; Ensor et al., 2011), there is a lack of data concerning the role of ToM (especially false-belief understanding) in modeling the relationship between EC and prosocial disposition. In the present study, conducted with a sample of preschoolers, we set out to address this gap by investigating how the relationship between children’s EC and prosocial orientation (a specific aspect of social competence) might be influenced by their false-belief understanding. Given that within the broader theoretical construct of social understanding, language is another key variable that contributes to explaining children’s cognitive and socio-emotional development, (Carpendale and Lewis, 2006) we also chose to include linguistic ability in our research design. In sum, the aim of our study was to add to previous knowledge about the relationship between EC and prosocial behavior by testing for the existence of possible mediational mechanisms involving language and ToM.

### Emotion Comprehension and Prosociality

Emotion comprehension is a set of abilities enabling the child to understand the nature and causes of emotions, and the fact that these may be regulated by using specific behavioral and cognitive strategies (Pons et al., 2004). Although children display a certain amount of individual variation in this regard, a substantial corpus of data has allowed the main steps in the development of EC to be identified (Pons and Harris, 2005; Harris, 2008). Specifically, children with a typical developmental profile progress through three main levels of EC referred to as *external, mental* and *reflective*, and comprising nine different components. Each transition from one level to the next represents an increase in the child’s ability to understand the effect of internal states on emotional experience.

Prosocial orientation is defined as an individual’s tendency to feel empathy for others and behave prosocially (Eisenberg et al., 2006). Empathy is the basic human ability to respond affectively to emotion in others and a prerequisite for many prosocial behaviors, where the latter are defined as a broad class of actions intentionally undertaken to benefit others (Zahn-Waxler et al., 1992). Positive social behavior first appears in early childhood, has been found to increase concurrently with EC throughout the preschool years, and is considered a marker of a personal orientation toward acting in a socially positive way (Eisenberg et al., 2006).

A number of studies have reported significant relationships between EC and specific aspects of prosocial orientation in preschoolers. For instance, Cassidy et al. (2003) found a strong correlation between 3- and 5-year-old children’s emotion understanding and their tendency to engage in cooperative play with peers and carry out prosocial actions. In addition, both Denham et al. (2003) and Eisenberg et al. (2006) found a correlation between preschoolers’ EC and their propensity to engage in actions that benefit others, such as sharing, helping or comforting. Ensor and Hughes (2005) also found that maternal ratings of prosocial behavior were positively correlated with performance in emotion-understanding tasks in very young children, even after controlling for the effect of age. Moreover, Garner et al. (2008) investigated associations between mother-child conversation about emotions and preschoolers’ social competence, highlighting that both mothers’ and children’s emotion explanations predicted prosocial behavior, but were negatively associated with aggressive actions. Similarly, Ensor et al. (2011) found strong associations between emotion understanding at 3 years and prosocial behavior at 4 years, even after controlling for the effect of verbal ability, which is known to be associated with both. In a longitudinal study with 3- to 5-year-olds, Eggum et al. (2011) found that emotion understanding was a predictor of children’s sympathy and prosocial orientation over time. More recently, Grazzani and Ornaghi (2015) and Ornaghi et al. (2015) found that training 4- to 5-year-old children in EC during a 6-week conversation-based intervention at school positively increased their prosocial orientation, even after controlling for gains in verbal ability. Interestingly, the positive effect was found to remain stable over time. Similar results were recently found by Grazzani et al. (2016) in a study with toddlers, showing that EC plays a crucial role in the development of even the earliest prosocial behavior.

### Emotion Comprehension and Prosociality: The Contribution of ToM

Theory of mind develops gradually throughout childhood, progressing from comprehension of the role of desire in behavior to include understanding of the role of beliefs and false beliefs (Wellman, 1990). Indeed the ability to solve false-belief tasks has become a key parameter for determining whether children are aware that other people may have perspectives that differ from their own. It is assumed that children have a ToM if they recognize that another person can hold a belief that is different from theirs and to the real state of affairs, and that the person will behave in a certain way on the basis of that belief.

Research on the association between EC and ToM in preschoolers has yielded partly conflicting results, in some cases suggesting that the two constructs are not correlated (Dunn, 1995), in others that they are positively correlated (Harwood and Farrar, 2006), and in still others that, though positively correlated, they represent distinct domains, given that once other variables such as age and language ability have been taken into account, they do not contribute to explaining each other (Cutting and Dunn, 1999).

In a longitudinal study by Dunn et al. (1991) the development of an understanding of mental states (ToM) was examined in relation to EC, where the latter was defined as the ability to label emotions and the ability to link particular situations to emotions. The authors found that patterns of family interaction at almost 3 years of age were more strongly associated with EC than with ToM when these abilities were assessed at 3 years and 4 months, leading them to hypothesize that EC and ToM follow distinct developmental paths. The finding that scores on ToM and EC tasks were not correlated with one another lent further support to this hypothesis. However, given the young age of the participants, these results were based on measures of ToM that did not include the classic FB tasks, but simpler tests on the relationship between beliefs and actions. In a later study, Dunn (1995) again found no correlation between EC and ToM in 40-month old children, a further indication of the importance of differentiating between these two domains in the study of social cognition.

However, in a subsequent longitudinal study with preschool children Hughes and Dunn (1998) found significantly related improvements in ToM tasks and affective perspective-taking, and stable individual differences over time. In follow-up research using a rich battery of tasks (which in the case of EC consisted of tasks assessing affective labeling and understanding of the links between particular situations and emotions), Cutting and Dunn (1999) investigated social understanding in preschoolers. In this case, they found a correlation between ToM and EC. Nonetheless, regression analyses suggested that the two domains were distinct and that variance in ToM did not contribute independently of other variables such as age, linguistic competence or family background (e.g., social class, size of family, family usage of internal state lexicon) to explaining variance in EC, or vice versa. The authors therefore concluded that their findings once again supported the hypothesis that, at least at around 4 years, the two domains are distinct and that the correlations found were due to other factors.

More recently, de Rosnay et al. (2004) and Bender et al. (2011) found positive correlations between the two domains in children ranging between 3 and 7 years. Similarly, Harwood and Farrar (2006) found ToM to be positively correlated with the ability to understand conflicting emotions. Finally, Weimer et al. (2012), in a study with 4½- to 6½-year-olds, found a correlation between comprehension of the external causes of emotion and ToM, adopting part of the same instrument used in this study to assess EC.

How might we explain these partially inconsistent findings across the different studies reported in the literature? A first consideration concerns methodological choices. For instance, given the young age of the participants, these results were sometimes based on measures of ToM that did not include classic FB tasks, but simpler tests on the relationship between beliefs and actions. This means that it is difficult to compare or aggregate the different results obtained, because the variables investigated were conceptualized and operationalized differently across studies (low construct comparability). Furthermore, the theoretical abilities explored in this line of enquiry are subject to constant and rapid development compared to other constructs. This means that the same measures cannot be used at different developmental stages, leading to further variation between datasets.

A second consideration is that certain points of convergence have begun to emerge among the existing studies and these may be identified and analyzed. First, all studies have viewed (and reported) EC and ToM as different but related constructs. In general, the relationship between the two constructs has been described as statistically significant, positive and weak in magnitude. From a longitudinal perspective, this relationship remains stable over time, meaning that both dimensions are crucial for coping with environmental challenges in a developmentally functional way (Whiten, 1999). Second, the fact that EC and ToM are viewed as distinct constructs implies the need for clearly stated hypotheses about possible “causal” relationships between EC and ToM to be clearly stated. In existing regression studies (Dunn et al., 1991; Weimer et al., 2012), the hypothesized relationship between EC and ToM is that the better a child’s understanding of emotion, the better its overall social cognition competence, with consequent improvement in its ability to differentiate between appropriate and inappropriate reactions, social interactions and behaviors. In sum, it seems that when children have an appropriate knowledge and understanding of emotions, their ability to comprehend the role of desire, belief and false belief in behaviors is more advanced.

With regard to prosociality, the contribution of ToM, especially false-belief understanding, to explaining children’s positive social behavior concurrently with EC has been documented. As reported in a very recent meta-analysis, many studies have shown that children with more advanced ToM abilities also receive higher scores on measures of prosocial behavior (Imuta et al., 2016). For instance, in a longitudinal study with 3- to 6-year-olds, Eggum et al. (2011) showed that ToM, as well as EC, predicted participants’ prosocial orientation as assessed via parent reports. In addition, Caputi et al. (2012) found significant correlations between 5- and 7-year-old children’ ToM and prosocial behavior, which in turn mediated the longitudinal effects of ToM on the quality of later peer relations. Finally, Walker (2005) examined the relationship between preschoolers’ false-belief understanding and teacher ratings of social competence, in terms of prosocial, aggressive and disruptive behavior. He reported that ToM scores significantly predicted prosocial behavior, especially for girls.

To sum up, the majority of studies have found ToM to correlate positively with both EC and prosociality. In a similar fashion, EC positively influences measures of prosocial behavior. This means that the network of relationships among the three constructs may be summarized as follows: EC represents the conventional starting point of a chain that sets off a cascade of direct and indirect effects on Tom and prosocial behavior. In other words, EC (as an antecedent) seems to affect false-belief understanding, which in turn influences prosocial behavior. Having reviewed these relationships, we can examine existing studies on the equally important role of language in relation to EC and prosocial behaviors.

### Emotion Comprehension and Prosociality: The Contribution of Language

The crucial role of language in children’s socio-emotional development has been documented by numerous studies with children of different age groups (de Rosnay et al., 2004; Milligan et al., 2007). Both cross-sectional (de Villiers and de Villiers, 2000; Astington and Baird, 2005) and training studies (Hale and Tager-Flusberg, 2003; Lohmann and Tomasello, 2003; Grazzani Gavazzi and Ornaghi, 2011; Ornaghi et al., 2011, 2014) have shown that language plays a key role in fostering children’s understanding of the mind.

Language abilities also contribute to the relationship between children’s social understanding skills and their social competence. For example, Cassidy et al. (2003) found that when preschoolers’ language ability was partialled out of the relationship between psychological understanding and positive social behavior, many of the significant correlations disappeared. Similarly, Ensor and Hughes (2005) found that emotion understanding and verbal ability explained over half the variance in maternal ratings of very young children’s prosocial behavior.

Furthermore, children with language impairments often experience difficulties with their social and emotional functioning and display poorly developed prosocial behavior (e.g., Farrar et al., 2009). Although recent studies have added to our understanding of this relationship, reporting that language ability plays a lesser role than previously believed (e.g., Bakopoulou and Dockrell, 2016), linguistic competence remains a crucial factor contributing to the development of both children’s psychological understanding and their social competence, especially prosocial behavior (e.g., Girard et al., in press).

In conclusion, language is another variable that can potentially influence the relationship between EC and prosocial behavior. It does so by providing social groups with a shared symbolic system (Bourdieu, 1991) on which to base communication. It thus facilitates children (as well as adults) in improving their social coordination (Malle, 2002) and prosociality (Farrar et al., 2009). For instance, language can make it easier for young children to express and communicate the meaning of their inner states to others. In addition, this process can help them to differentiate among emotions because they learn that different words reflect different states (e.g., happy, enthusiastic). At the same time, via the systematic exchange of feedback with the environment and other people (i.e., via language as a symbolic communication system), children improve their competence in recognizing others’ emotional states and responding with appropriate behaviors. As previously mentioned, this mechanism has been corroborated by studies with children affected by language impairments who often experience difficulties with their socio-emotional functioning (e.g., Farrar et al., 2009). Children with an advanced understanding of others’ cognitive and mental – and not only affective and emotional – states generally have a greater appreciation of the difficulties and situational factors affecting others, which may induce feelings of empathy and sympathy and as a consequence foster prosocial behavior (Eggum et al., 2011).

### The Present Study

As discussed above, many longitudinal studies have investigated the effects of EC on the development of social competence, showing it to be a key predictor of prosocial behavior from the preschool years through the end of childhood. Other studies have shown that both ToM, as assessed via false-belief tasks, and language ability may facilitate children’s disposition to act prosocially toward others. Nonetheless, as far as we are aware, no studies have examined the cumulative role of false-belief understanding and language in modeling the relationship between EC and prosocial behaviors. To address the lack of data in this area, a series of multiple regression analysis (mediation analysis) were performed to test how and to what extent the relationship between EC and prosocial orientation is influenced by false-belief understanding and language ability. As emphasized by Stone (1992), it is of paramount importance to focus on the evaluation of mediators in order to address the mechanisms by which certain effects occur.

With this aim in mind, we expected EC to be positively related to prosocial orientation. In fact, a growing corpus of data, primarily collected with preschoolers, shows that EC is a key correlate and predictor of prosociality from early childhood (e.g., Cassidy et al., 2003; Denham et al., 2003; Ensor and Hughes, 2005; Eisenberg et al., 2006). We also expected that EC would be positively related to false-belief understanding. Many studies have found a significant relation between these two important social understanding competences (e.g., de Rosnay et al., 2004; Harwood and Farrar, 2006; Weimer et al., 2012). Finally, we expected that EC would be positively related to language ability. Numerous studies have shown a significant relationship between children’s EC and their language abilities (Ensor and Hughes, 2005). We thus expected to find similar results to the international literature in our sample of 101 Italian preschoolers and thereby to contribute by replicating evidence from other studies (between-sample cross-validation). In addition, given that we were interested in developing a cumulative model of the relationships among all the variables under study, we used mediation analysis to test the following hypothesis: that the relationship between EC and prosocial orientation would be fully mediated by participants’ levels of false-belief understanding and language ability.

The proposed mediational model suggested a chain of relations such that the direct effect of EC on prosocial behavior would tend to disappear when variations in ToM and language ability were taken into account. We are referring here to the assessment of natural direct effects (Pearl, 2001) which allows for natural variation between subjects in the level of the mediators. Please note here that, if the data supports the hypothesis, full mediation implies that when low scores are obtained for the mediator variables (M_1_, M_2_ →0), the relationship between EC and prosocial behavior will be absent. In other words, if children do not have adequate language abilities and an appropriate understanding of false beliefs they will tend to be less prosocially orientated, regardless of their level of EC.

To control for sources of covariation within the mediation model, both age in months and gender were included as covariates. This meant analyzing the segregated data given that (1) this procedure is more specific and informative (Pepe and Addimando, 2014; Pearl et al., 2016) and (2) it is a means of controlling for the Yule-Simpson effect (i.e., a statistical association that holds for the full sample but is reversed in all sub-populations, Simpson, 1951). The added value of this overall approach is that it allows us to explore whether an effect can be decomposed into direct and mediated components (Alwin and Hauser, 1975).

The results of this study were intended to contribute to our understanding of how, we should go about fostering positive qualities and behaviors in children, with a view to enhancing our communities and families, and our children’s abilities to live responsible and dignified lives in these settings (Seligman and Csikszentmihalyi, 2000). Extending what is known of the network of relations among EC, prosocial orientation and false-belief understanding means learning more about the crucial ability to get along with other people in everyday life (Grazzani Gavazzi et al., 2011). In the final section of the article, we examine both the present study’s limitations and its implications for designing practical intervention to enhance children’s development.

## Materials and Methods

### Ethics Statement

The research was conducted according to the APA ethical standards, and was approved by the local Ethics Committee.

### Participants

Participants were 101 preschool children (54 boys), ranging in age between 50 and 75 months (*M* = 62.4 months; *SD* = 7.04). All spoke Italian as their first language, and no child had any known language impairment, learning problems, or psychological difficulties. The children were recruited at five kindergartens in Northern Italy on the basis of informed consent obtained from their parents. Neither socioeconomic data nor ethnic information were explicitly collected; however, the schools the participants attended served predominantly middle-class families, very few of whom were of non-Italian origin.

### Measures

The following measures of verbal ability, false-belief understanding, EC and prosocial orientation were administered individually in counterbalanced order. They were administered at school by trained researchers following the ethical principles and code of conduct of APA (American Psychological Association, 2010).

#### Peabody Picture Vocabulary Test (PPVT, Dunn and Dunn, 1981)

The Italian standardized version of the test (Stella et al., 2000) was used. It evaluates the receptive vocabulary of children between 3 and 12 years and consists of 180 cards, each containing four numbered illustrations among which the child is asked to indicate the one that corresponds to the word called out by the examiner. Scoring was carried out following the standard procedure, with 1 point assigned for each correct answer and 0 for each wrong answer. The reliability coefficient was α = 0.71.

#### False-Belief Understanding Battery

Participants were administered a false-belief location change task (the ‘Sally and Ann test’ by Baron-Cohen et al., 1985) and an Italian adaptation of the false-belief explanation task developed by Peskin and Astington (2004). In the first task, children were asked to predict where a story character would look for an object that had been moved from one place to another without her knowledge, and were assigned a score of 1 for the correct answer and 0 for a wrong answer. The second task consisted of four illustrated scenarios presented in counterbalanced order to assess participants’ conceptual understanding that a story character may be ignorant of a situation the participant knows to be true. Children were asked to provide a false-belief explanation for each of the four stories. Each explanation was awarded a score of 1 for a spontaneously provided appropriate explanation using a metacognitive term (e.g., *know, think, wonder*) or a term implying a mental state (e.g., *see, tell, check*), 0.5 for an appropriate explanation requiring a prompt, or 0 for any other reply or failure to respond. The reliability coefficient for the explanation task was α = 0.60. Scores for the battery were summed, yielding a possible maximum total score of 5 (4 for the explanation task and 1 for the location change task).

#### Test of Emotion Comprehension (TEC, Pons and Harris, 2000)

We used the standardized Italian version of the test devised by Albanese and Molina (2008). The TEC evaluates, between the ages of 3 and 11, children’s developing understanding of the nature, causes and regulation of emotion in terms of nine components of EC (recognition of facial emotional expressions, the impact of situational causes on emotion, the role of desires in emotion, the role of beliefs in emotion, the impact of memory on emotions, the effect of morality on emotions, awareness that emotions may be regulated, understanding that there may be a discrepancy between manifest and felt emotions, and appreciation of concurrent mixed emotions) distributed across external, mental and reflective levels of development. The researcher reads very short illustrated stories to the child and asks control questions in relation to the more complex scenarios to ensure that the child has understood them. After each story, the child is asked to indicate how the protagonist really feels in this situation by choosing one of four faces representing different emotional states. For each of the nine components, children obtained a score of 0 for a wrong answer and a score of 1 for choosing the correct option. By summing the correct answers, a total score ranging from 0 to 9 was obtained. In addition, we calculated partial scores for the three levels, each ranging from 0 to 3. Data displayed satisfactory reliability, α = 0.69.

#### The Prosocial Orientation Story-Completion Task (Ornaghi et al., 2015)

This instrument consists of four illustrated scenarios, presented in counterbalanced order, which describe familiar situations encountered by a story character (a boy or girl, in line with the gender of the individual participant), and followed by a question about the story that is designed to assess the respondent’s prosocial orientation. The four items focus on specific prosocial behaviors: comforting, peacemaking, sharing, and helping. The researcher reads the story and, after a control question testing the child’s comprehension of the story (“What happened here?”), asks her to say how it ends (**Table 1** shows the items of the task and some examples of children’s prosocially oriented answers). Participants’ responses were audiotaped and transcribed. Following the scoring procedure in Ornaghi et al. (2015), a score of 0, 0.5, or 1 was awarded for each item, yielding a maximum possible score of 4. The data were independently coded by two judges who attained a satisfactory level of inter-rater reliability, Krippendorff’s α = 0.82. Furthermore, the instrument displayed convergent validity with both the ToM (*r* = 0.45; *p* < 0.0001), and the EC (*r* = 0.39; *p* = 0.001) measures.

**Table 1:** Examples of children’s prosocially oriented answers to the items of the prosocial orientation story-completion task.

Target behavior	Scenarios of the task	Examples of participants’ prosocially oriented answers
COMFORTING	Andrew is going to school with Albert. Albert is crying because he tripped on a stone and fell and hurt himself.	*“Andrew helps Albert to pick himself up.”*
	How do you think the story will end?	*“When they get to school, Andrew runs to tell the teacher so she puts a plaster on the part that is hurting him and he stops crying.”*
PEACEMAKING	Andrew is at the playground with Luke and Elliot. There is only one swing and both Luke and Elliot want to go on it. Andrew sees them begin to quarrel.	*“ehm… that Andrew helps them to calm down and they don’t fight any more.”*
	How do you think the story will end?	*“Andrew says they can take turns, that way both of them are happy and they make friends again.”*
SHARING	Andrew is in the school yard with her classmates. Andrew sees Leo snatch the ball from John. John starts to cry.	*“Andrew tells Leo to give the ball back to John. He’s got to ask before taking it. Like: Can I play with the ball too?”*
	How do you think the story will end?	*“Andrew tells Leo that instead of snatching the ball they can all play together.”*
HELPING	Andrew sees that Lucy does not know how to draw a car. Andrew is very good at drawing because his Dad taught him.	*“Andrew goes over to Lucy and tells her that he will help her draw the car.”*
	How do you think the story will end?	*“Andrew tells Lucy that if she wants, he’ll show her how to draw the car, then she’ll be able to draw it too.”*


### Procedure for Mediation Analysis

Generally speaking, a mediation model assesses whether the relationship between two variables (a determinant variable -X- and a criterion variable -Y-) remains unvaried when a third variable (the mediator -M-) is taken as accounting for the relationship between them (Preacher et al., 2007; Veronese and Pepe, 2014).

**FIGURE 1:**
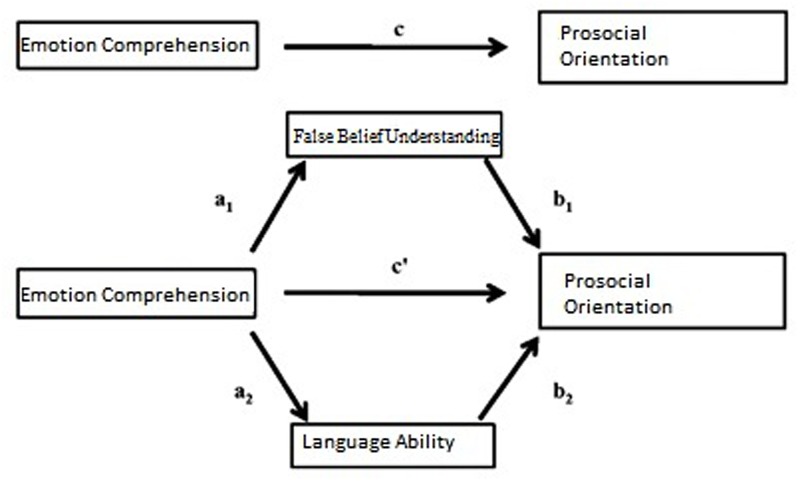
**Hypothesized multiple mediation model**.

Following in the mediation studies tradition, our research design evaluated whether and to what extent false-belief understanding (M1) and language ability (M2) influenced (mediated) the relationship between EC (X) and prosocial orientation (Y) (see **Figure 1**). The mediating variables occupy an intermediate position in the sequence of relations between EC and prosocial orientation. Thus, mediation is defined here as a generative mechanism in which the effect of Y on X is transmitted by M. Mediation also implies a temporal relation among variables, with X occurring before M and M occurring before Y. To control for sources of covariation within the mediation model, both age in months and gender were included as covariates in Preacher and Hayes’ macro. In this case, age and gender were taken as variables that might be related to EC and prosocial behaviors and that “might falsely accentuate or obscure the relation between them” (Meinert, 1986, p. 285).

The analytical procedure for testing mediation models is well-established in the social sciences; there are three requirements (Baron and Kenny, 1986) for a model to be accepted: (1) that changes in the determinant variable account for changes in the mediators (see **Figure 1**, paths a_1_ and a_2_); (2) that changes in the mediators account for variation in the criterion variable (see **Figure 1**, paths b_1_ and b_2_); and, finally, (3) that the statistically significant relationship between the determinant and the criterion variable (see **Figure 1**, path c) is no longer statistically significant (c’) when paths a_i_ and b_j_ are controlled for the mediating variables. The last of these conditions (variations in c paths) has recently been debated in the social science context (Wu and Zumbo, 2007), given that some scholars tended to interpreted loss of statistical significance with reduction in magnitude of the c’ path as a “partial mediation.” On the contrary, it is today widely agreed that if c’ is no longer statistically significant, the mediation model should be accepted without invoking the concept of *partial mediation* (Hayes, 2013). The testing strategy for mediation is based on repeated step-wise regression analyses and it requires different regression equations (Hayes, 2013). Given that it is desirable to control the mediation model for multiple potential sources of covariation, in the present study the mediation analysis was conducted using Preacher and Hayes (2008) macro for SPSS 22. The macro is useful for dividing the size of the total effect of X on Y into two parts: the direct effect of X on Y and the indirect effect of X on Y through a mediator M (Hayes, 2009). In addition, a bootstrap non-parametric resampling procedure was applied. Bootstrapping procedures can compensate for the limitations of statistical methods that assume standard distribution (for details see Shrout and Bolger, 2002) in small to moderate sample sizes (*N* < 500, Hoyle and Kenny, 1999). Thus, bootstrap analysis with 5,000 bootstrap sample simulations was conducted to obtain estimates of the indirect effects along with their 95% confidence intervals. Finally, in order to correct for type I error (Loeys et al., 2015) and potential dependence among direct and indirect effects (Tofighi et al., 2009), the Bonferroni correction for statistical significance was applied and, as a consequence, *p* was set at 0.025.

All variables used during the mediation test were centered to enhance the interpretability of the data and reduce potential issues linked to multicollinearity. The data were also checked for assumptions related to multiple regression (i.e., multivariate normality). No violations to normality were found. Finally, a criterion of *p* < 0.001 for Mahalanobis’ distance was applied: one single multivariate outlier was identified and skipped. The final sample was composed of 100 participants.

## Results

The results are presented in two sections. First, descriptive statistics for all measures and zero-order correlations between variables are given. Second, the outcomes of the mediation test and bootstrapping procedure are reported.

### Descriptive Statistics and Correlational Analyses

**Table 2** shows means, standard deviations, skewness, and asymmetry values for all the measures under study. Preliminary examination of the measures suggested that all indicators were essentially normally distributed. Distribution values were good with no violation of the most stringent cut-off point of 1 for skewness (Tabachnick and Fidell, 2013) and only a slight violation for kurtosis.

**Table 2:** Descriptive statistics.

	Mean	Standard deviation	Skewness	Kurtosis
Emotion comprehension	4.71	1.34	-0.218	0.036
False-belief understanding	2.12	1.36	-0.064	-1.07
Prosocial orientation	1.47	1.19	0.532	-0.639
Language ability	64.83	20.25	-0.057	-0.440


*standard error for skewness was 0.241.*

**Table 3** shows the zero-order correlations among the variables included in the model. Correlation values were generally found to be statistically significant and in the theoretically expected directions. The variable age was significantly correlated with all the other variables (language ability, EC, false-belief understanding, and prosocial orientation). Similarly, language ability showed strong positive correlations with EC, false-belief understanding and prosocial orientation. EC was highly correlated with prosocial orientation, and false-belief understanding was found to have positive correlations with both emotion understanding and ToM. **Table 4** reports partial correlations among EC, false-belief understanding, language ability and prosocial orientation after age and gender had been controlled for. Overall, analysis of the correlations supported the viability of the multiple mediation analysis.

**Table 3:** Zero-order correlations among variables.

	1	2	3	4	5
Age in months (1)	–				
Language ability (2)	0.550^∗∗^	–			
Emotion comprehension (3)	0.451^∗∗^	0.532^∗∗^	–		
False-belief understanding (4)	0.273^∗∗^	0.456^∗∗^	0.484^∗∗^	–	
Prosocial orientation (5)	0.319^∗∗^	0.357^∗∗^	0.436^∗∗^	0.454^∗∗^	**–**


*^∗∗^*p* < 0.01.*

**Table 4:** First-order correlations partialled out for children’s age and gender.

	1	2	3	4
Language ability (1)	–			
Emotion comprehension (2)	0.386^∗∗^	–		
False-belief understanding (3)	0.381^∗∗^	0.420^∗∗^	–	
Prosocial orientation (4)	0.234^∗^	0.229^∗^	0.304^∗∗^	–


*^∗^*p* < 0.05, ^∗∗^*p* < 0.01.*

### Mediation Analyses

We next assessed a multiple mediation model in which false-belief understanding and language ability were hypothesized to mediate the relationship between EC and prosocial orientation.

**FIGURE 2:**
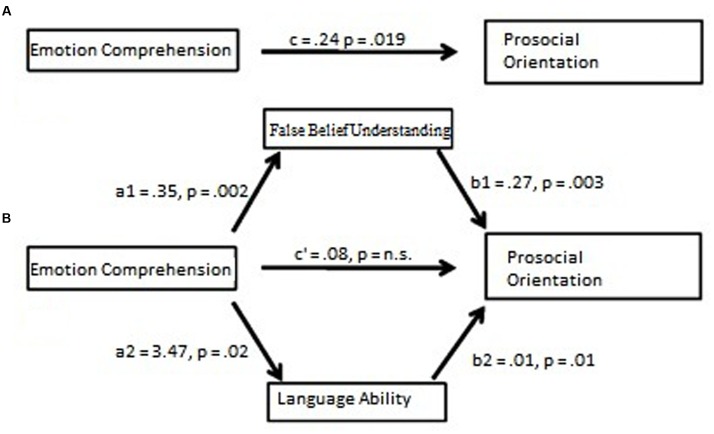
**(A)** The effect of EC on prosocial orientation **(B)** The effect of EC on prosocial orientation via the two proposed mediators: false-belief understanding and language ability.

As reported in **Figure 2**, when the total direct effect of EC on prosocial orientation was assessed via the proposed mediators, the c’ path was no longer statistically significant. EC had a direct positive effect on both FBU, *a1* = 0.35, *p* = 0.002, and language ability, *a2* = 3.47, *p* = 0.019. Bootstrap analysis of the indirect effect of false-belief understanding on the path between EC and prosocial orientation suggested a bias corrected 95% confidence interval that did not include the zero value, CI [0.0233,0.225]. Similar analysis of the indirect effect of language ability on the relationship between EC and prosocial orientation pointed to a bias corrected 95% confidence interval that did not include the zero value, CI [0.011,0.164]. Analysis of the covariates revealed that gender did not play a statistically significant role, β = 0.15, *p* = 0.47, while children’s age had a trivial positive effect, β = 0.03, *p* = 0.04). These results, along with the statistical significance of the remaining paths confirmed the hypothesis of a multiple mediation model that accounted for 36% of the variance in prosocial orientation, *R^2^* = 0.36; *F*(5,94) = 12.44, *p* = 0.0001.

## Discussion

The present research investigated the potential mediating effects of constructs related to ToM in the relationship between EC and prosocial orientation. To our knowledge, this is one of the few studies to have quantified and analyzed, using a multiple mediation model, the role of emotional, cognitive and linguistic factors in explaining children’s prosocial orientation. More specifically, it investigated the association between EC and prosocial orientation in a sample of preschoolers with a view to addressing the question – still unresolved in the literature – of how this relationship is influenced by ToM and language, both key components of social understanding along with EC and prosocial disposition. Thus, the core aim of the present study was to advance our understanding of the role of ToM and language in promoting prosocial orientation among preschool children, as well as to seek greater clarity regarding the relationship between EC and prosocial orientation. On the basis of previous findings on the link between EC and prosocial orientation, and on their respective correlations with both ToM and language, we had hypothesized that the relationship between EC and prosocial orientation would be fully mediated by false-belief understanding and language ability.

In line with the outcomes of a large number of studies with preschoolers (e.g., Eisenberg et al., 2006; Eggum et al., 2011; Ornaghi et al., 2015), which have shown that EC is a key indicator and predictor of prosociality from early childhood, we found that participants’ EC and prosocial orientation were highly positively correlated. Additional evidence for this link comes from recent intervention studies showing that training young children in EC skills (such as understanding the expression and causes of emotion or the fact that emotions may be regulated) fosters their disposition to behave prosocially toward others and helps them to better recognize and understand other people’s emotional states and needs (Aram and Shlak, 2008; Ornaghi et al., 2015; Grazzani et al., 2016).

In line with the existing literature, our data also confirmed that false-belief understanding and language ability are both significantly correlated not only with children’s EC (Harwood and Farrar, 2006; Ornaghi et al., 2014) but also with their disposition to act prosocially (Cassidy et al., 2003; Walker, 2005; Caputi et al., 2012). Within the broad framework of social understanding, many researchers have demonstrated that children’s EC and ToM performance affect their interpersonal behavior making them more prosocial toward adults and peers. Similarly, our findings are in line with a number of studies proving that language ability is a consistent correlate of children’s comprehension of epistemic and non-epistemic inner states (Milligan et al., 2007) as well as of their prosocial behavior (Durkin and Conti-Ramsden, 2007). In this regard, although some studies have reported no relationship with language, much empirical evidence strongly suggests taking language into account as a potential mediating or moderating variable influencing the association between socio-emotional and cognitive competences (Imuta et al., 2016).

Confirming our hypothesis, false-belief understanding and language were both found to play a positive and significant role in influencing the relationship between EC and prosocial orientation. Those findings represent an original contribution to existing knowledge about relations within the theoretical framework of social understanding components, by identifying ToM and language as two key mediators. With regard to the contribution of ToM, it is well established that when children become aware of the thoughts, intentions and beliefs of others, they are better able to link other people’s emotion and feelings to their intentions and behavior. In particular, an advanced understanding of the cognitive states of others allows children to accurately discern an interlocutor’s difficulties in a given situation, and this in turn may produce feelings of empathy and foster prosocial behavior (Cassidy et al., 2003; Eggum et al., 2011). With regard to language ability, as mentioned above, it offers children a valuable tool with which they can participate in social interactions, conversational exchanges, pretend play, story-telling and other activities that foster their perspective taking, in terms of their ability to link their own and others’ manifest actions with mental states (Nelson, 2007). In this way, language represents a crucial ability that helps children to make explicit and share their own and others’ inner states, such as feelings, thoughts and needs. A key line of enquiry for future follow-up research will be to explore what aspects of language (e.g., syntax, pragmatics) make the greatest contributions to explaining children’s individual differences in prosocial disposition.

On close examination of the fully mediated relationship between the variables, the leading contribution of ToM and language ability in explaining children’s prosociality identified in this study means that observed differences in prosocial behavior are essentially not explained by EC but by ToM skills and language. In conclusion, it appears that the inclination to behave prosocially in recognition of other people’s needs is more influenced by preschoolers’ ability to comprehend others’ beliefs and false beliefs and by their language abilities than by their comprehension of emotion.

### Study Limitations

The main limitations of the present research should be acknowledged and discussed. First, a crucial pre-requisite of any mediation model is that the mediating variables “precede” the outcome variable (Baron and Kenny, 1986). Given that the design of this study is cross-sectional, the temporality of the relationships among EC, false-belief understanding, language ability and prosocial orientation cannot be empirically resolved. As a consequence, the results of the present study should be not read in terms of causation or co-causation. Nevertheless, the analysis of the existing literature suggests that EC influences prosocial orientation (Eggum et al., 2011; Ornaghi et al., 2015) and that both language ability and false-belief understanding are factors involved in determining levels of prosociality (Cassidy et al., 2003; Ensor and Hughes, 2005; Walker, 2005). In this regard, future studies adopting a longitudinal approach are required to thoroughly and meaningfully evaluate potential causal directions among EC, prosocial orientation and their mediators.

Second, we gathered data from a relatively small sample (*N* = 100) of preschoolers, and this reduced the power of the statistical tests, and the possibility to generalize from the findings. With regard to the former aspect, in quantitatively analyzing the data, every effort was made to offset the limited sample size (bootstrapping procedure, confidence intervals, macro for multiple mediation, evaluation of sources of co-variation). However, concerning generalizability, future research with additional samples of children at different ages and in different socio-economic contexts is needed in order to further confirm the mediation model and chain of relationships identified here.

### Educational Implications

Despite the above mentioned limitations of the study, the current findings suggest the value of involving children, from early childhood onward, in educational activities designed to enhance language ability and false-belief understanding skills in the direction of improving prosocial orientation in children rather than focusing exclusively on their EC competence (Brandt et al., 2016). The implementation of practical training of this kind, perhaps as a form of early (or ongoing) intervention in educational settings, may contribute to fostering positive qualities and behaviors in the participating children, thereby enhancing their ability to live responsible and dignified lives in the context of their community (Seligman and Csikszentmihalyi, 2000; Majorano et al., 2009).

Children who display more advanced EC and ToM skills will also be more inclined to engage in prosocial behavior aimed at benefiting others. In turn, this kind of positive behavior will influence the quality of children’s later relationships with peers (Caputi et al., 2012). In designing relevant educational intervention, adults and educators should take due account of the crucial role of language as the cultural and social tool *par excellence* (Vygotskij, 1934) in enabling cognitive and socioemotional development.

## Author Contributions

VO gave substantial contribution to the design of the work, the acquisition and interpretation of data, and the draft of the manuscript. AP gave an important contribution to the data analyses and their interpretation. IG gave a substantial contribution to the design of the work and the discussion of the outcomes.

## Conflict of Interest Statement

The authors declare that the research was conducted in the absence of any commercial or financial relationships that could be construed as a potential conflict of interest.
